# Exploration of the Potential Mechanism of Tao Hong Si Wu Decoction for the Treatment of Breast Cancer Based on Network Pharmacology and *In Vitro* Experimental Verification

**DOI:** 10.3389/fonc.2021.731522

**Published:** 2021-08-26

**Authors:** Shi Huang, Yan Chen, Lingyu Pan, Changyi Fei, Ni Wang, Furui Chu, Daiyin Peng, Xianchun Duan, Yongzhong Wang

**Affiliations:** ^1^The First Affiliated Hospital of Anhui University of Chinese Medicinee, Hefei, China; ^2^College of Pharmacy, Anhui University of Chinese Medicine, Hefei, China

**Keywords:** Taohong Siwu Decoction (THSWD), network pharmacology, breast cancer, traditional Chinese medicine, target identification

## Abstract

**Background:**

Tao Hong Si Wu Decoction (THSWD) is a well-known traditional Chinese medicine used clinically alone or combined with drugs to treat breast cancer. However, there has been no study to date on the underlying mechanisms of its therapeutic effects.

**Objectives:**

To explore the potential mechanism of THSWD for the treatment of breast cancer using network pharmacology and experimental research.

**Methods:**

The active ingredients of THSWD were screened according to Lipinski’s rule of five based on the 107 ingredients of THSWD identified by UPLC-Q-TOF-MS^E^. The targets of THSWD and breast cancer from multiple databases were collected, and a Compound-Target-Pathway network based on protein-protein interaction (PPI) was constructed. Gene ontology (GO) analysis and KEGG pathway analysis were performed *via* the DAVID server. Molecular docking studies verified the selected key ingredients and key targets. The results of network pharmacology were verified by *in vitro* experiments. Including the effects of THSWD drug-containing rat serum (THSWD serum) on cell proliferation, and on the targets HRAS, MAPK1, AKT1, GRB2, and MAPK14 were assayed by RT-qPCR and Western blot assays.

**Results:**

In total, 27 active ingredients including 8 core components, were obtained from 107 ingredients and 218 THSWD target genes for the treatment of breast cancer were identified. THSWD is active in the treatment of breast cancer by targeting Ras, FoxO, PI3K-Akt and other signaling pathways. MCF-7 and MDA-MB-231 cell proliferation was inhibited by THSWD serum in a time and concentration dependent manner. THSWD could regulated the RNA and protein expression of core targets HRAS, MAPK1, AKT1, GRB2, and MAPK14 for treatment of breast cancer.

**Conclusion:**

The results of network pharmacology study showed that THSWD is active against breast cancer by intervening with multiple targets and pathways. Luteolin, kaempferol, senkyunolide E, and other 8 compounds may be the core active ingredients of THSWD in the treatment of breast cancer. THSWD treatment of breast cancer may be related to targeting Ras, FoxO, PI3K-Akt, and other signal pathways associated with the core targets HRAS, MAPK1, AKT1, GRB2, and MAPK14.

## Introduction

The latest data from the International Agency for Research on Cancer (IARC) survey showed that Female breast cancer has surpassed lung cancer as the most commonly diagnosed cancer, with an estimated 2.3 million new cases (11.7%) in 2018 (1). Epidemiological data across Chinese cancer centers in 2019 showed that the incidence and mortality rates of breast cancer were first and fifth among female malignant tumors, respectively, and both rates have shown a rising trend. Breast cancer has become a serious disease that threatens the health of women in China and women across the world. At present, the most common clinical treatment interventions are surgical resection, radiotherapy, and chemotherapy, but surgical resection does not improve prognosis, while long-term radiotherapy and chemotherapy can cause serious side effects. Studies have shown that the combined treatment with traditional Chinese medicine (TCM) can improve the quality of life of breast cancer patients (2). Therefore, it is of great significance to search for the TCM treatment of breast cancer and to explore the prevention and treatment of breast cancer.

Tao Hong Si Wu Decoction (THSWD) was documented in “The Golden Mirror of Medicine” compiled by Wu Qian in the Qing Dynasty. It consists of six species of medicinal herbs: Prunus persica (L.) Batsch (Taoren, TR), Carthamustinctorius L. (Honghua, HH), Angelica sinensis (Oliv.) Diels (Danggui, DG), Ligusticum chuanxiong Hort (Chuanxiong, CX), Paeoniae Radix Alba (Baishao, BS), and Rehmannia glutinosa (Gaertn.) DC (Shudi, SD). THSWD eliminates blood stasis and promotes blood circulation and nourishment (3). Xia using network pharmacology and *in vivo* experiments, found that THSWD plays a therapeutic role in postpartum blood stasis by regulating oxidative stress through the mitochondria (4). THSWD promotes angiogenesis after cerebral ischemia in rats through activation of platelets (5). Further, THSWD may inhibit tumor angiogenesis in breast cancer patients by decreasing microvessel density (MVD) and vascular endothelial growth factor-A (VEGF-A) expression (6). Clinically, it is often added or subtracted on the basis of this prescription to treat cerebrovascular, gynecological cardiovascular and others, DG and CX have good therapeutic effects on gynecological conditions, especially breast diseases. Our group previously combined UPLC-Q-TOF-MS^E^ technology to investigate the therapeutic effects of THSWD on breast cancer in a murine model and revealed its beneficial effects were closely related to apoptosis (7). However, research on the mechanism by which THSWD functions is still not fully clear, therefore, it is of great theoretical value and research significance to further explore the mechanism of action of THSWD in the treatment of breast cancer. Network pharmacology is an emerging discipline that combines pharmacology, computer science, and information networking on the basis of systems biology (8). Through network pharmacology, data on ingredients, diseases, and their related targets are often gathered through existing online databases, and visualization software is then used to systematically analyze TCMs by constructing an “ingredient-target-pathway” network, so as to define the drug intervention and the treatment effects on different diseases (9). The systematic nature of network pharmacology is consistent with the holistic view and syndrome differentiation theory of TCM has been widely applied to the study of TCM to generate new research data, methods and results (10–12). Based on this approach, we used network databases to construct an “ingredient-target” network, a PPI network for enrichment analysis. Next, we used molecular docking studies to further investigate the mechanisms through which THSWD exerts its therapeutic effects and these were accompanied by *in vitro* cell experiments, to provide a basis for its clinical application in breast cancer.

## Materials and Methods

### Network Pharmacology Study

#### Screening of Active Ingredients of THSWD

The *in vitro* chemical composition of THSWD was determined by ultraperformance liquid chromatography quadrupole time of flight mass spectrometry (UPLC-Q-TOF-MS^E^) (7), and compounds were selected according to the Lipinski’s rule of five (13)— MWT ≤ 500, H-bond donors ≤ 5, H-bond acceptors ≤ 10, and logP ≤ 5—to select the most likely active ingredients. Moreover, several active ingredients with relative high content or excellent bioactivity, which did not satisfy these criteria, were also manually supplemented as candidate compounds for further analysis (14).

#### Compound-Related Targets Prediction

The mol2 file or SDF file of the active ingredient was downloaded using the zinc website (zinc.docking.org) and PubChem website (pubchem.ncbi.nlm.nih.gov) and was uploaded to the PharmMapper database (www.lilab-ecust.cn/pharmmapper) and “Homo sapiens” was selected for the prediction results.

#### Breast Cancer-Related Targets Prediction

The terms for “breast cancer”, for breast cancer targets and the OMIM database (http://omim.org) and the Genecards database (http://www.genecards.org) search.

#### Intersection of Compound Targets and Breast Cancer Targets

After obtaining the targets related to THSWD and targets of breast cancer, overlapping targets were selected. The overlapping targets are the possible targets for THSWD to exert therapeutic effects on breast cancer, which can improve the accuracy of screening targets and increase the credibility of the results. Using the “Compounds-targets” network of Cytoscape 3.7.1 software, the ingredients and intersection targets are nodes in the network, and the edges represent connections between ingredients and their active targets. The network was topologically analyzed using the “network analyzer” function of the software. The key compounds of THSWD for breast cancer treatment were screened based on the analysis results.

#### Establishment of the Protein-Protein Interaction Network

Common targets were uploaded to the STRING database (https://www.string-db.org/) to construct a protein-protein interaction (PPI) network. Protein species were set as human origin, and minimum required interaction score was set as the “highest confidence (0.900)” while concealing free points, to obtain the PPI network. The PPI network data were input into Cytoscape 3.7.1 software, and the PPI network was analyzed using the “network analyzer” function, with nodes whose degree and betweenness centrality values were greater than those of mean values as the key targets. In the network, node means compound or target, high degree indicates greater probability that the compound or target is exerting its effect.

#### Gene Ontology and Pathway Enrichment Analysis

Gene Ontology (GO) functional enrichment and KEGG pathway enrichment were performed using the David database (https://david.ncifcrf.gov/), in which the GO analysis included 3 components: Biological Process (BP), Cellular Component (CC), Molecular Function (MF), and the False Discovery Rate (FDR) values were set to obtain GO and KEGG enrichment results, we used the cluster profiler R package to perform GO analysis and KEGG analysis for the potential targets of THSWD in the treatment of breast cancer.

#### Molecular Docking Validation of Key Components and Key Targets

The top three key compounds from the results of item “2.1.4” were output as mol2 format files in TCMSP database (https://tcmspw.com/tcmsp.php), and the top three key targets in the PPI network, in the PDB database (http://www.rcsb.org) were used to define the molecular structures. Molecular docking of key active ingredients with key targets was performed using Autodock VINA software, binding energies were evaluated as evaluation indexes, and PyMOL software was used to present the results.

### *In Vitro* Experiments

#### Chemicals and Reagents

Prunus persica (L.) Batsch (Taoren, TR, batch number: 17033101), Carthamus tinctorius L. (Honghua, HH, batch number: 17041401), Angelica sinensis (Oliv.) Diels (Danggui, DG, batch number: 16070501), Conioselinum anthriscoides ‘Chuanxiong’ (syn. Ligusticum chuanxiong Hort) (Chuanxiong, CX, batch number: 17061601), Paeoniae lactiflora Pall. (Baishao, BS, batch number: 17050301), and Rehmannia glutinosa (Gaertn.) DC (Shudi, SD, batch number: 17042501) were purchased from Anqing Huashi Chinese Herbal Medicine Co. Ltd. (Anqing, China). All TCM materials were qualified by Professor Huasheng Peng (hspeng@126.com). For cell culture studies, Dulbecco’s modified Eagle’s medium (DMEM) was purchased from Gibco and fetal bovine serum (FBS) were purchased from Gemini(Shanghai China).

#### Sample Preparation

The herbs TR, HH, DG, SD, CX, and BS (3:2:3:4:2:3) were soaked then decocted twice with 10 volumes of 75% ethanol for 2 h and 8 volumes of 75% ethanol for 1.5 h. The extraction solutions were then filtered and combined. The filtrates were concentrated to 18 g/kg.

#### Animals

Healthy male adult Sprague-Dawley (SD) rats weighing 200 ± 20 g and aged 63–70 days were purchased from Shandong Experimental Animal Center (permit number: scxk-2019000). After adaptive feeding for 7 days, 25 rats each were randomly assigned to the rat serum control group and THSWD serum groups using a random numbers table.

#### THSWD Serum

Rats were administered the THSWD solution (9.0g/kg) intragastrically by gavage once daily for 7 days. Rats in the blank group were given saline. Blood samples were obtained from the aorta 1 h after the last gavage, all rats were anesthetized by intraperitoneal injection of chloral hydrate (350 mg-kg^-1^) and sacrificed by cervical vertebrae dislocation. The serum was separated from the blood. Subsequently, the serum was put in water bath 56°C for 30 min to be inactivated, after which the bacteria were eliminated by filtering through a 0.22-μm filter and stored at -20°C until use. All experiments were subject to approval by the Committee on the Ethics of Animal Experiments of Anhui University of Chinese medicine (Permit Number: AHUCM-Rats-2021023).

#### Cell Culture

Human breast cancer MCF-7 (Cat: 100137) and MDA-MB-231 (Cat: 339911) cell lines were obtained from Beina Biology (Beijing China), maintained in RPMI 1640 media supplemented with 10% FBS and 1% penicillin/streptomycin, then incubated in a humidified atmosphere at 37°C with 5% CO_2_. The media was changed daily and logarithmic growth cells were recorded for the experiment.

#### Cell Proliferation Assay

We seeded MCF-7 and MDA-MB-231 cells in a 96-well plate. The results of network pharmacology show that THSWD exerted therapeutic closely to Ras related Signaling pathways. GRB2, AKT1, MAPK1 and MAPK14 were located up/downstream of ras pathway. Lonafarnib (SCH-66336), is a potent and orally active farnesyl transferase (FTase) inhibitor that has shown anticancer activity. Lonafarnib inhibits the activities of H-RAS, K-RAS and N-RAS. The experimental conditions were divided into the blank control, negative control group, different concentrations of THSWD serum, and different concentrations of the inhibitor lonafarnib. Cells were incubated for 24 h, 48 h, and 72 h with the different concentrations of THSWD serum (1.25%, 2.5%, 5%, 10%, 20%, 40%, 50%), and different concentrations of the inhibitor Lonafarnib (1, 2.5, 5, 10, 20, 25, 30 μM). CCK-8 was added to each well, and cells were incubated for 2 h, and then the optical density at 450 (OD450) of each well was detected by enzymatic-reader (BioRad 680). There were six replicates for each treatment.

#### RNA Isolation and Real-Time Quantitative Polymerase Chain Reaction

Total RNA was extracted with Trizol solution following the manufacturer’s instructions. cDNA was synthesized with a first strand cDNA synthesis kit. SYBR Green qPCR SuperMix was used for real-time quantitative polymerase chain reaction (qRT-PCR). The reaction conditions were 50°C 2 min; 95°C 2 min; 95°C 15 s, and 60°C 32 s, for 40 cycles; melting curve analysis was at 60°C–95°C. Each sample was assayed three times by qRT-PCR (Biorad IQ5). Relative mRNA expression was normalized to the corresponding β-actin expression and analyzed by the 2^−△△Ct^ method. The nucleotide sequences of the primer pairs used for Quantitative gene expression are provided in **Table 1**.

**Table 1:** The nucleotide sequences of the primer pairs used for Quantitative gene expression.

Gene name	Forward and Reverse Primer (5'to3')	Amplicon length
HRAS-F	5'-TGCCATCAACAACACCAAG-3'	143
HRAS-R	5'-CCTGCCGAGATTCCACA-3'	
MAPK1-F	5'-CCAGAGCAAGTCCTCCAG-3'	130
MAPK1-R	5'-GGCACCAACAGTACAAAGC-3'	
AKT1-F	5'-AAGCCCCAGGTCACGTC-3'	116
AKT1-R	5'-TCGCTGTCCACACACTCC-3'	
GRB2-F	5'-CTGGAGCGTTTGCTGTG-3'	131
GRB2-R	5'-CCAGGTGTAGAATGCCAGA-3'	
MAPK14-F	5'-CACAGGGCCACCTTCTT-3'	100
MAPK14-R	5'-GCACCTCCCAGATTGTCTT-3'	
beta-Actin-F	5'-TCTCCCAAGTCCACACAGG-3'	127
beta-Actin-R	5'-GGCACGAAGGCTCATCA-3'	

#### Western Blotting

RIPA lysate buffer (Beyotime Biotechnology, P0013B) containing 1% PMSF was used to extract the total proteins from cells, after SDS-PAGE, the target protein range was mapped according to a marker size position. The separated proteins were then transferred to nitrocellulose filter membranes. The membrane was blocked overnight at 4°C in TBS-Tween 20 (TBST) buffer containing 5% skimmed milk powder. The HRP‐labeled secondary antibody was used after washing membranes in TBST and was incubated with secondary antibodies for 1.5 hour at room temperature. Finally, the protein bands were imaged using an enhanced chemiluminescence system (P90720, Millipore).

#### Statistical Analysis

SPSS 23.0 software was used for statistical analysis. The results were expressed as mean ± standard deviation (x ± sd). Multiple groups of independent data were compared using single factor analysis of variance. Pairwise comparisons between multiple groups were performed using t-test. *P*-values were calculated to show statistically significant differences. P < 0.05 was considered statistically significant.

## Results

### THSWD Active Ingredients

Of the 107 ingredients of the THSWD, 27 active ingredients were screened *in vitro* using the Lipinski’s rule of five (shown in **Table 2**), including 3 from Taoren, 8 from Honghua, 6 from Danggui, 2 from Shudi, 4 from Baishao, and 10 from Chuanxiong. Among these, there were 5 common components between Danggui and Chuanxiong, 2 common components between Baishao and Honghua, and 1 common component between Shudi, Baishao and Honghua.

**Table 2:** Active components of THSWD.

ID	Active Component	MW	nOHNH	nON	miLog P	Source
MOL001320	Amygdalin	457.48	7	12	-2.30	T
MOL001321	Mandelonitrile	133.16	1	2	1.20	T
MOL000771	P-Hydroxycinnamic acid	164.17	2	3	1.64	T
MOL002690	Hydroxysafflor-yellow-A	612.59	12	16	-4.45	H
MOL000340	3-Phenylpropionic acid	150.19	1	2	1.93	H
MOL002737	Scutellarein	342.34	0	6	3.10	H
MOL000006	Luteolin	286.25	4	6	2.07	H
MOL000415	Rutin	610.57	10	16	-1.45	H
MOL004474	4-Ethylphenol	122.18	1	1	2.51	H
MOL000414	Caffeic acid	180.17	3	4	1.37	D
MOL001456	Citric acid	192.14	4	7	-1.39	D
MOL010858	Tianshic acid	330.52	4	5	3.67	D
MOL002111	Butylidenephthalide	188.24	0	2	3.00	D C
MOL002143	Senkyunolide C	204.24	1	3	2.74	D C
MOL002146	Senkyunolide F	206.26	1	3	1.84	D C
---	Jionoside B1	814.78				S
MOL000360	Ferulic acid	194.20	2	4	1.62	S B H
MOL001935	paeonilactone B	196.22	1	4	-0.10	B
MOL000422	Kaempferol	286.25	4	6	1.77	B H
MOL000513	Gallic acid	170.13	4	5	0.63	B H
MOL011782	Ligustilide	190.26	0	2	2.94	C
MOL002208	Senkyunolide A	192.28	0	2	3.19	C
MOL002209	Senkyunolide G	208.28	1	3	2.54	C
MOL000114	Vanillic acid	168.16	2	4	1.15	C
MOL002181	4-Hydroxy-3-Butylphthalide	206.26	1	3	2.99	C
MOL002049	Ferulaldehyde	178.20	1	3	1.67	C D
MOL011786	Senkyunolide E	204.24	1	3	1.90	C D

### Common Targets of THSWD and Breast Cancer

**Figure 1:**
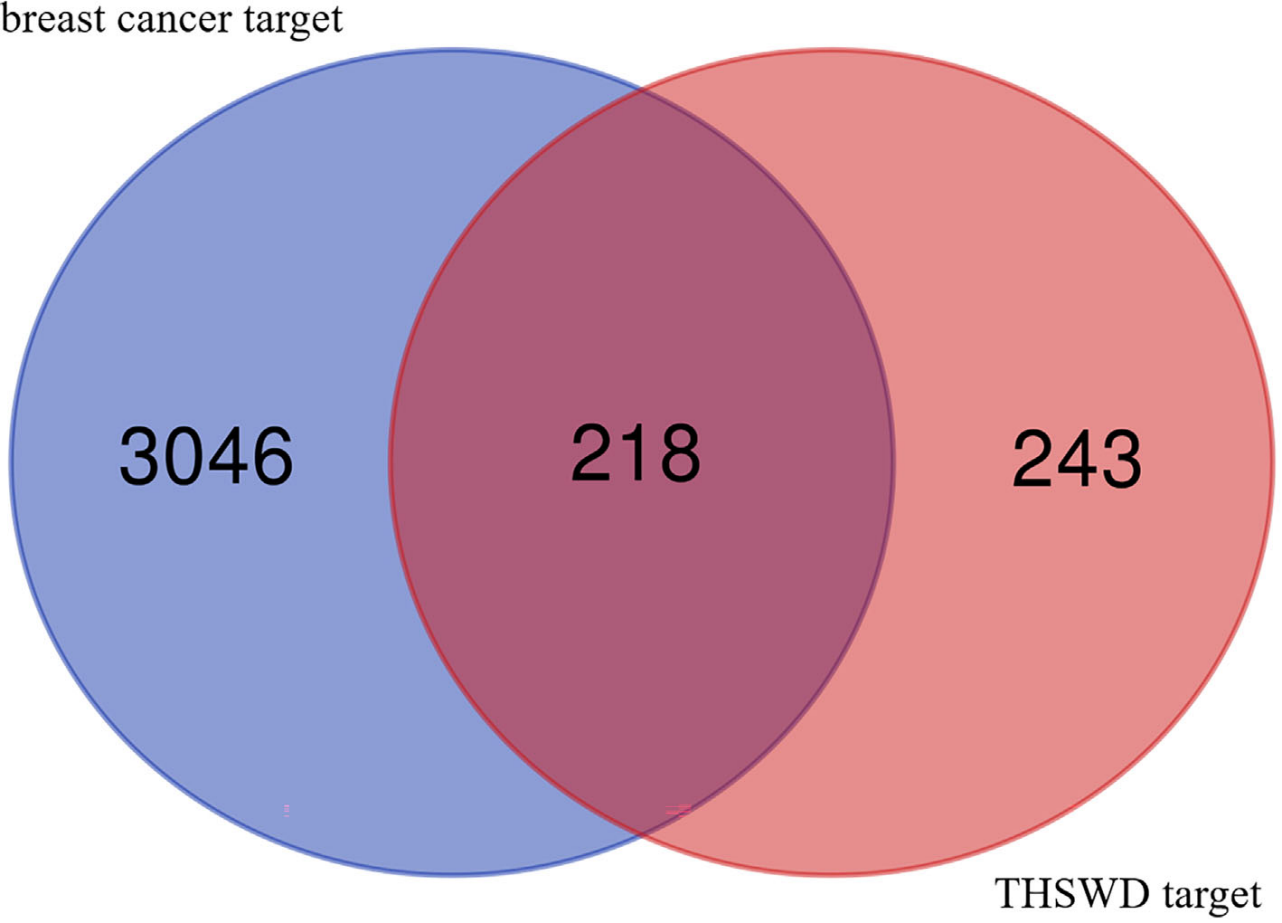
Venn diagram of component target and disease target.

Integration of the targets of the ingredients in the PharmMapper database produced 5757 gene targets. After removing duplicates, a total of 461 relevant human genes were obtained. In OMIM and Genecard databases, 3264 repetitive breast cancer-related targets were obtained, the 461 targets predicted with the active ingredients of THSWD were intersected using a Venn diagram, resulting in 218 intersection targets, which were potential active targets of THSWD acting on breast cancer (**Figure 1**).

### “Components-Targets” Network Analysis

**Figure 2:**
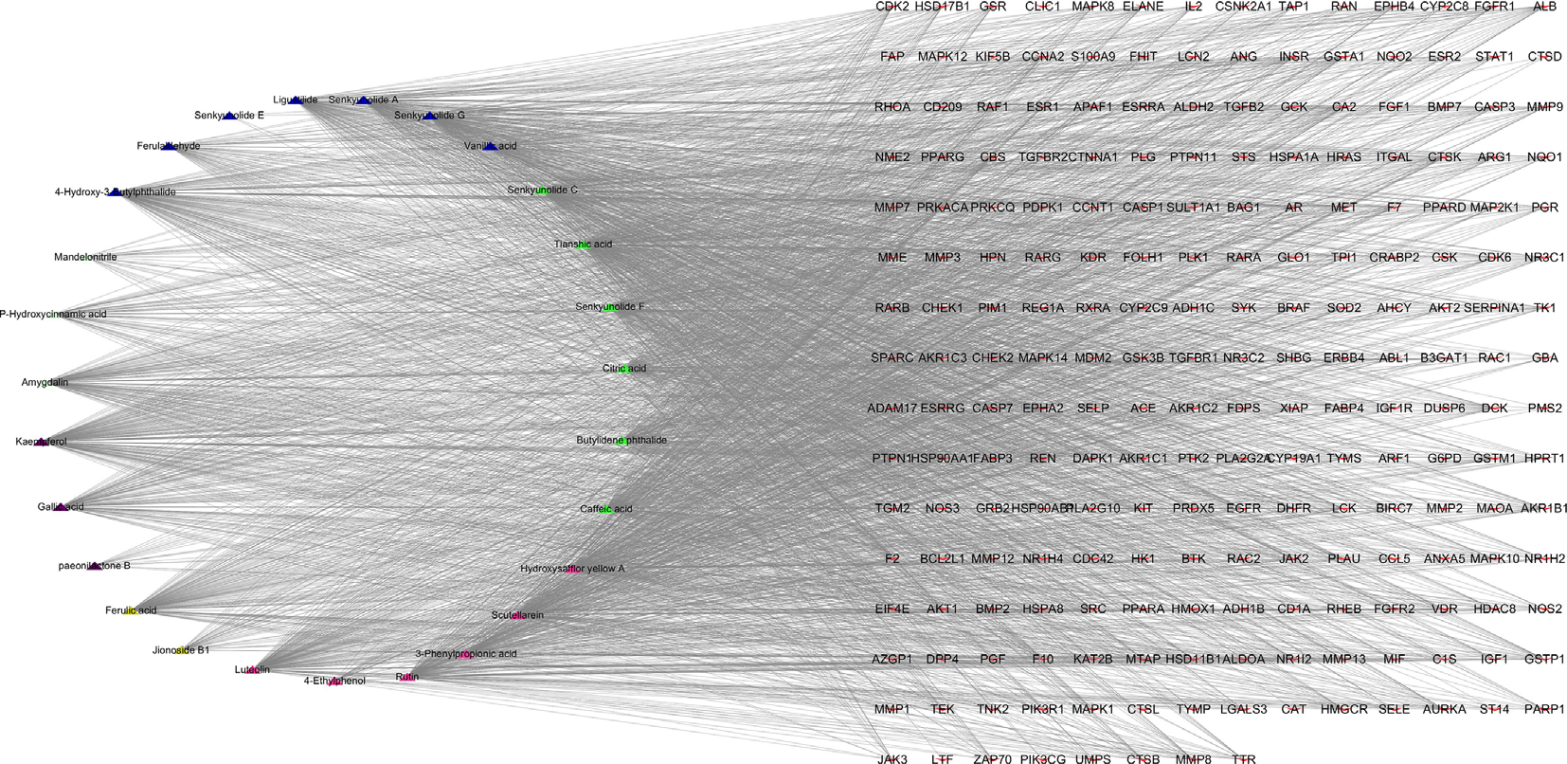
“Components-targets” interaction network.

Twenty-seven active components and 218 common targets were imported into Cytocsape 3.7.1 software to construct the “Components-targets” network. There were 245 nodes (27 active ingredients and 218 targets) and 2855 edges in the network, shown in **Figure 2**. The network analyzer results showed, the average degree of node was 105.74, the average betweenness centrality was 0.034, the average closeness centrality was 0.511, and there were 11 compound nodes whose values all exceeded the average value (shown in **Table 3**), it is speculated that these compounds may be key compounds for THSWD to exert therapeutic effects against breast cancer.

**Table 3:** Basic information of hub compounds of Tao-Hong-Si-Wu Decoction in the treatment of breast cancer.

Compound	Degree	Betweenness Centrality	Closeness Centrality
Senkyunolide E	166	0.11965001	0.65498652
Kaempferol	158	0.06122716	0.62790698
Luteolin	157	0.05458365	0.62467866
Rutin	152	0.07629322	0.60902256
Scutellarein	151	0.05224282	0.60598504
Senkyunolide C	148	0.07755585	0.5970516
Hydroxysafflor yellow A	148	0.05995414	0.5970516
4-Hydroxy-3-Butylphthalide	147	0.05197661	0.59413203
Senkyunolide G	146	0.04863749	0.59124088
Ferulic acid	134	0.04116615	0.55862069
Senkyunolide A	128	0.03593588	0.54362416

### PPI Network Analysis

**Figure 3:**
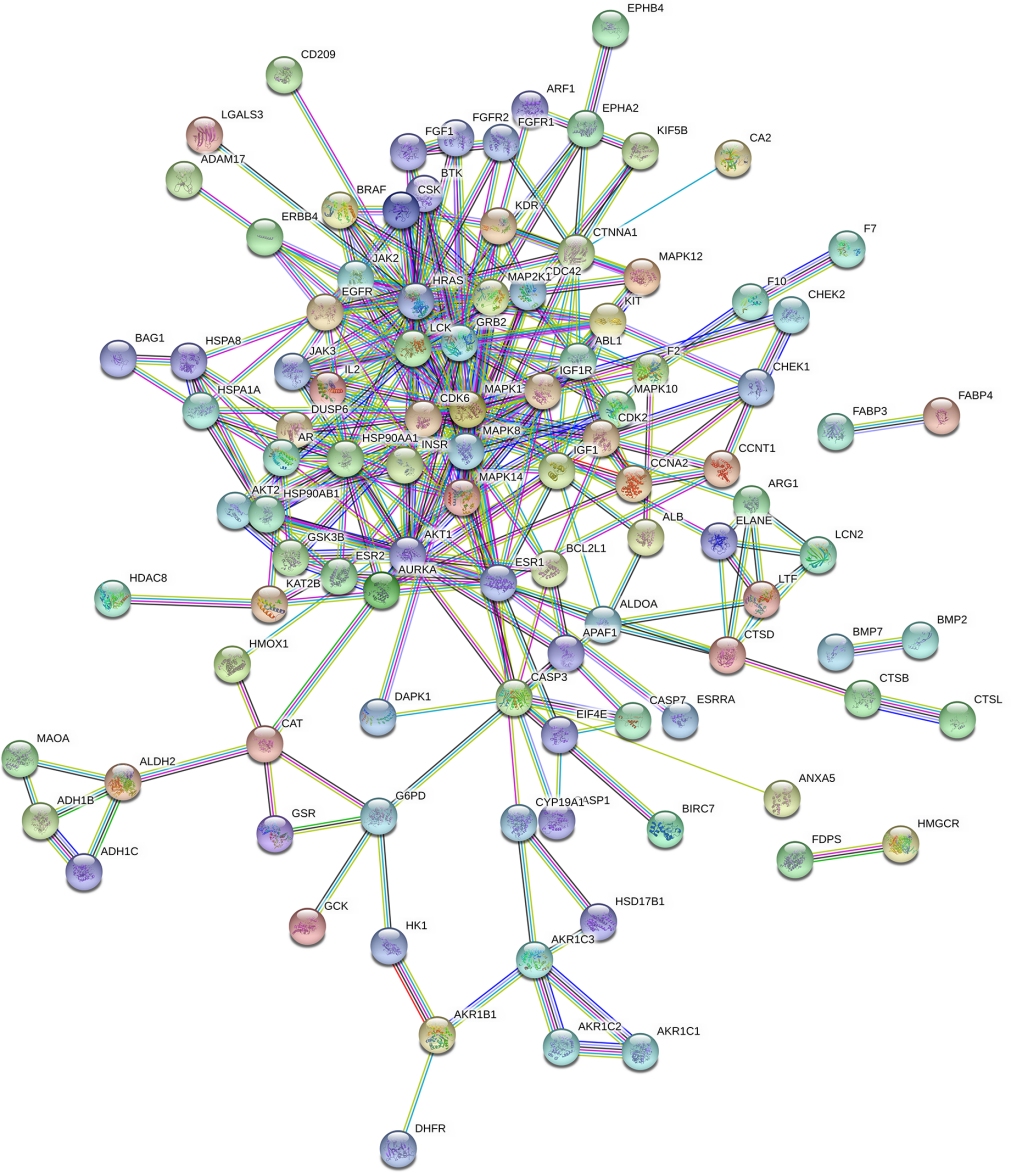
PPI network of THSWD in treating potential targets of breast cancer.

**Figure 4:**
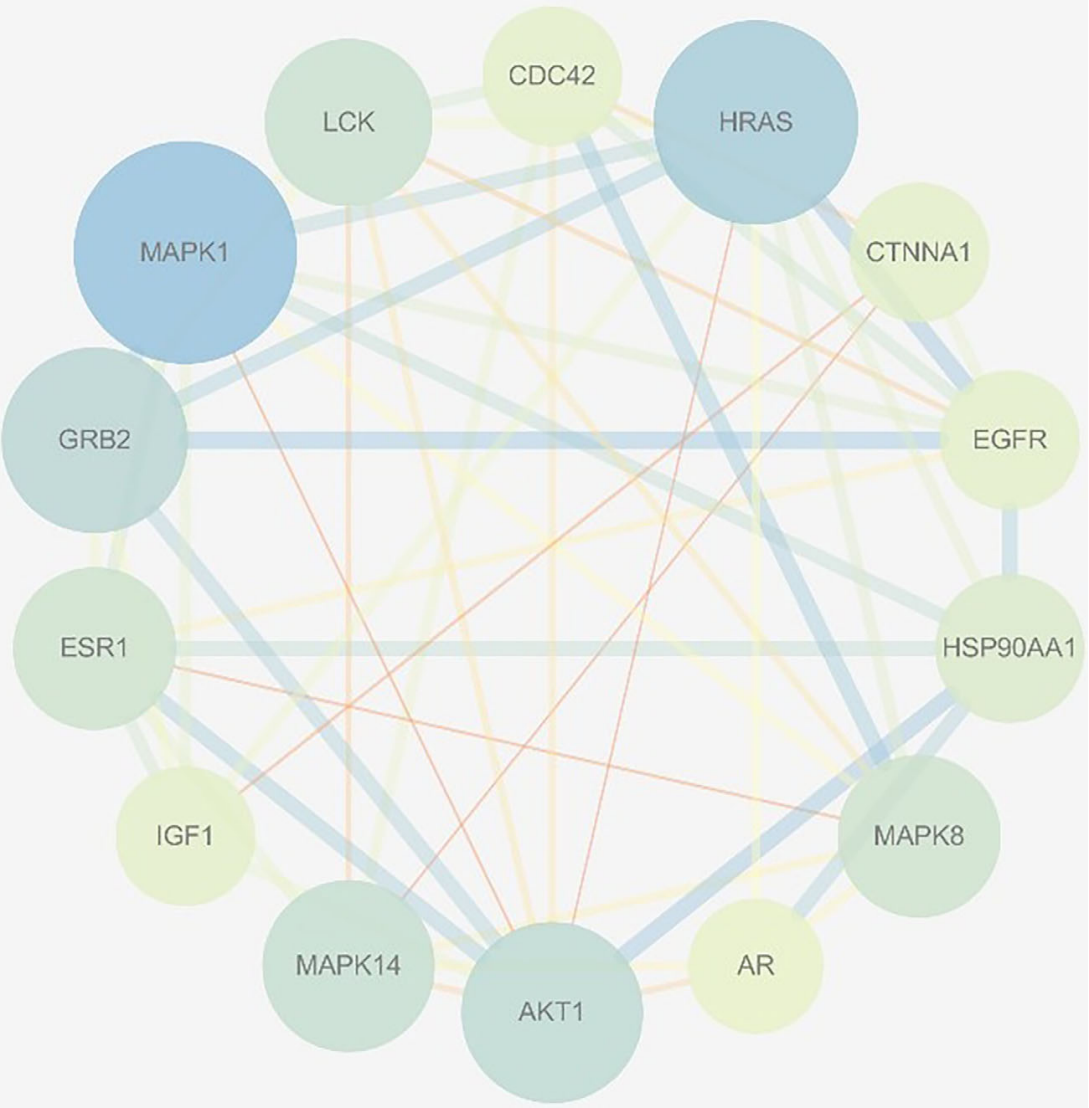
Selected 14 core targets.

In total, 102 nodes and 301 edges were included in the PPI network, indicating that 102 targets could interact with each other, resulting in a total of 301 interactions, shown in **Figure 3**. The network analyzer results showed, the average degree of nodes was 5.902, the average betweenness centrality was 0.022, and the average closeness centrality was 0.367; there were 14 compound nodes whose values all exceeded the average value, It was visualized and displayed by Cytoscape software, in which, node size represented the size of degree value, and the larger the node, the greater the corresponding degree value, and the higher the degree of interaction association of this protein with others, We speculated that these targets were be the key targets for THSWD for the treatment of breast cancer (**Figure 4**).

### GO Function Enrichment Analysis

**Figure 5:**
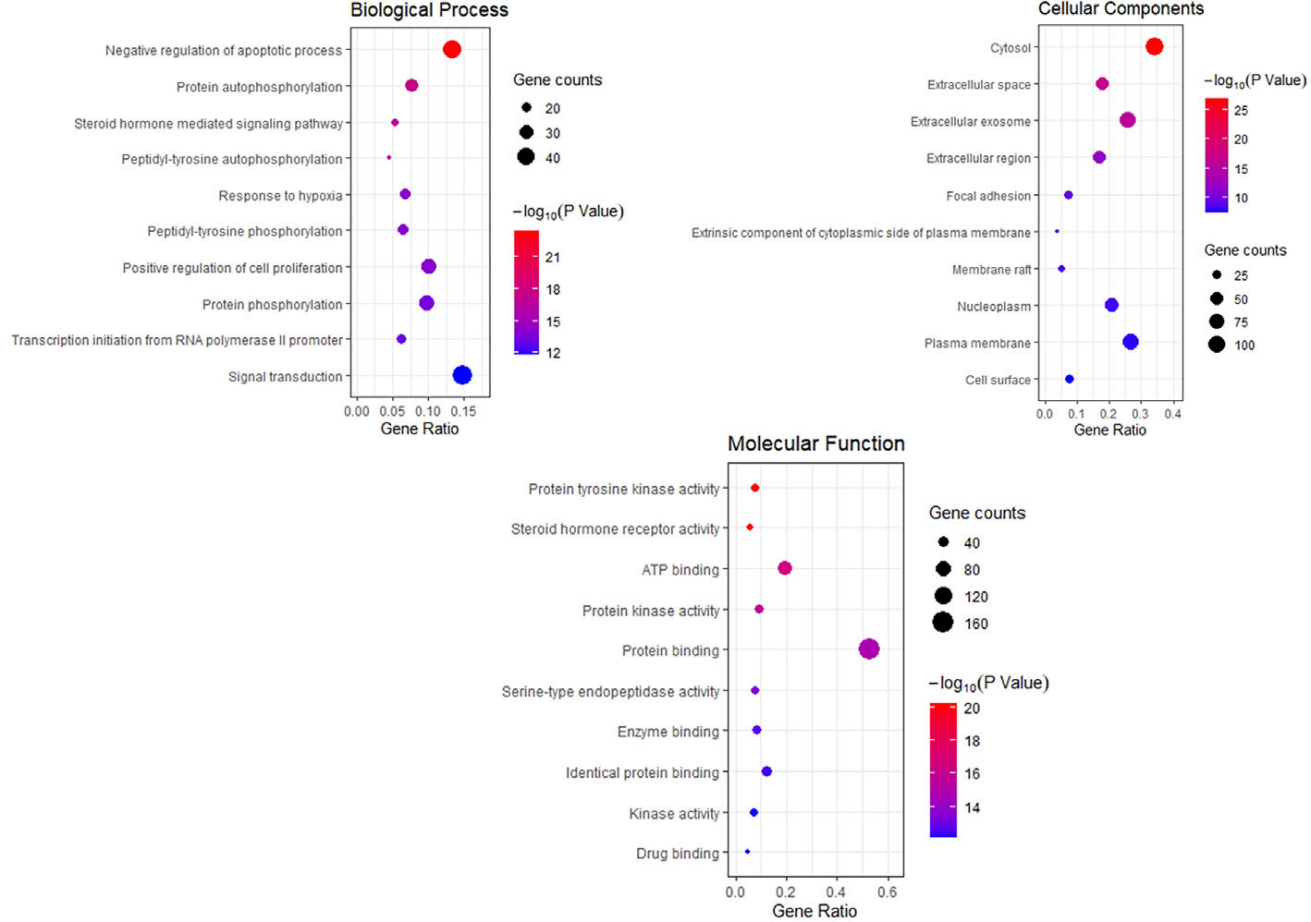
Bubble Diagram of GO function enrichment of potential targets from the THSWD for Treatment of breast cancer. The larger the number of enriched targets, the larger the dots; the larger the P value, the bluer the dot color.

To explore the dynamic activity of THSWD in breast cancer, we used the DAVID database to enrich the GO bioprocesses of 218 common targets; FDR and P-values <0.01 were used for filtering. The results of the GO enrichment analysis contained 62 BPs involved in negative regulation of apoptotic processes, protein autophosphorylation, steroid hormone-mediated signaling pathways, and peptidyl-tyrosine autophosphorylation; 32 MF involved protein tyrosine kinase activity, steroid hormone receptor activity, and ATP binding; 16 CC involved cytosol, extracellular space, and extracellular exosomes. The top 10 related entries for BP, MF, and CC are shown in bubble plots using the cluster profiler R package (**Figure 5**).

### KEGG Analysis

**Figure 6:**
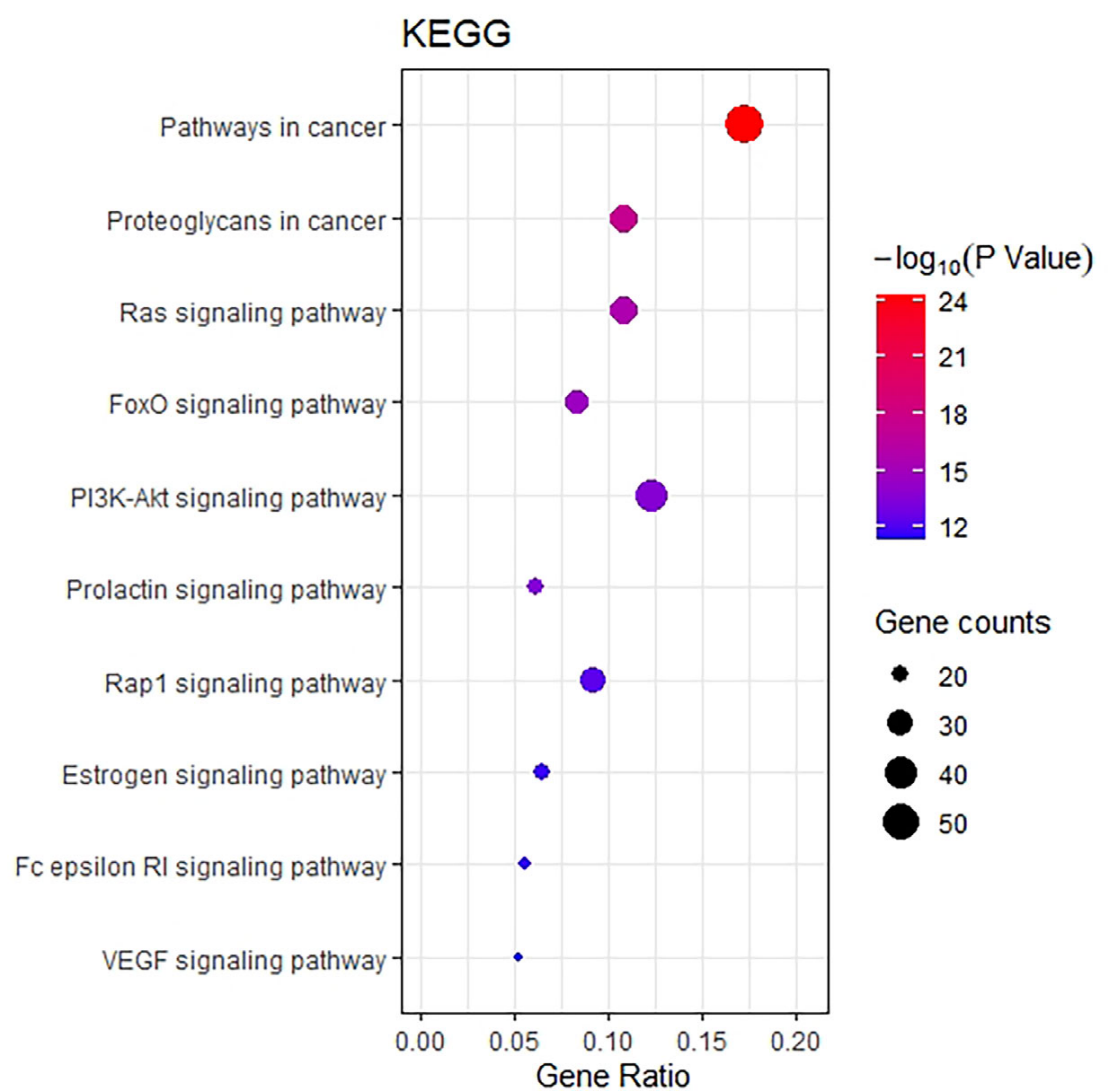
Bubble Diagram of KEGG enrichment of potential targets from the THSWD for treatment of breast cancer. The larger the number of enriched targets, the larger the dots; the larger the P value, the bluer the dot color.

The KEGG pathway enrichment function of pathways module in David database was used to explore the function of 218 potential gene targets in signaling pathways involved in the treatment of breast cancer by THSWD. KEGG enrichment analysis showed a total of 54 pathways with significant differences (FDR<0.01, P<0.01). The top 10 pathways are visually represented in a bubble plot in **Figure 6**. KEGG enrichment results indicated that THSWD might exert therapeutic effects by participating in the regulation of RAS, FOXO, PI3K/Akt, and other signaling pathways.

### THSWD-Prescription Composition-Active Ingredients-Targets-Pathway

**Figure 7:**
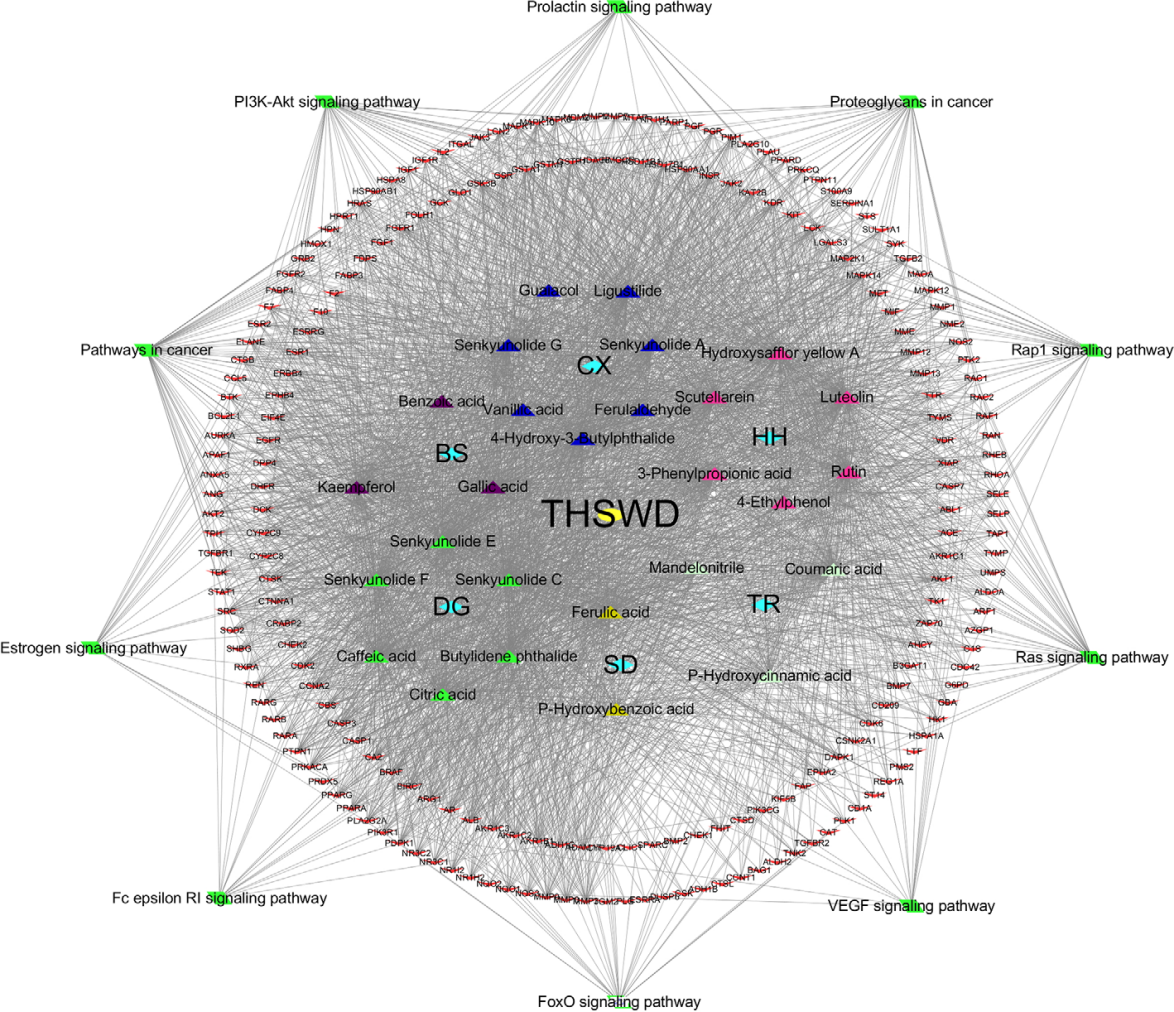
“THSWD-prescription composition-active ingredients-targets-pathways”. The yellow diamond represent THSWD, the cyan diamond represent medicinal herbs, the triangle represent the components, the red v-shaped represent the intersection targets, and the green diamond represent the signal paths.

Based on the KEGG pathway enrichment results, the top 10 ranked signaling pathways that were identified to be closely related to breast cancer were combined with information of the THSWD active ingredients and intersection targets with breast cancer to construct a “THSWD-prescription composition-active ingredients-targets-pathway” network (**Figure 7**). This plot intuitively indicates that the processes of THSWD for the treatment of breast cancer involve multiple active ingredients, targets, and pathways.

### Molecular Docking Results

**Figure 8:**
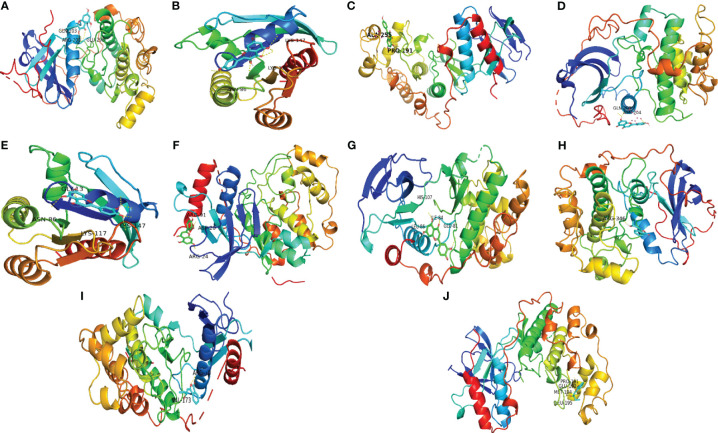
Schematic diagram of docking results. **(A)** Kaempferol-AKT1; **(B)** Kaempferol-HRAS; **(C)** Kaempferol-MAPK14; **(D)** Luteolin-AKT1; **(E)** Luteolin-HRAS; **(F)** Luteolin-MAPK1; **(G)** Luteolin-MAPK14; **(H)** Senkyunolide E-AKT1; **(I)** Senkyunolide E-MAPK1; **(J)** Senkyunolide E-MAPK14 ARG, arginine; GLN, glutamine; LYS, lysine; GLY, glycine; ASP, aspartic acid; HIS, histidine; LEU, leucine; GLU, glutamic acid; VAL, valine; ALA, alanine; PRO, proline; ILE, isoleucine.

The top 3 ingredients selected from 11 key ingredients with the corresponding top 5 of 14 key targets were subjected to molecular docking, and the results are shown in **Table 4**, and the partial docking conformations are shown in **Figure 8**. Different ligand molecules have greater affinity for the receptor protein through hydrogen bonding. The molecular docking results demonstrated that the key active ingredients of THSWD could tightly bind to the relevant targets, confirming the ingredient target prediction results. The binding energies between key components and corresponding target proteins did not exceed -4.5 kcal/mol, which indicated that the key components of THSWD had good binding ability to the corresponding target proteins. Most of the docked targets were distributed in pathways involving cancer, such as Ras, FoxO, PI3K-Akt, and other signaling pathways, thus demonstrating that the active ingredients of THSWD could have an impact on the occurrence and development of breast cancer by acting on the related targets and their corresponding signaling pathways.

**Table 4:** Molecular docking results.

Molecule Name	Target Name	Docking score (kcal/mol)
Kaempferol	AKT1	-5.8
Kaempferol	HRAS	-5.4
Kaempferol	MAPK14	-7.6
Luteolin	AKT1	-6.1
Luteolin	HRAS	-5.9
Luteolin	MAPK1	-6.7
Luteolin	MAPK14	-8.0
Senkyunolide E	AKT1	-4.9
Senkyunolide E	MAPK1	-5.3
Senkyunolide E	MAPK14	-7.5

### *In Vitro* Experiments

#### THSWD Serum and Inhibitor Lonafarnib Effects on Breast Cancer Cell Proliferation

**Figure 9:**
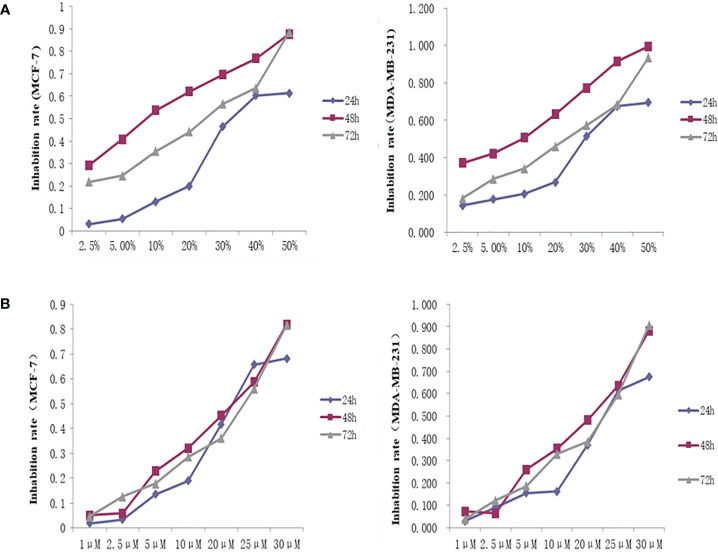
Inhibition curve of different concentrations of THSWD serum and inhibitor Lonafarnib on MCF-7, MDA-MB-231 cells. **(A)** THSWD serum **(B)** inhibitor Lonafarnib.

The OD450 values and inhibition rate of cells in each group were calculated by treating the cells with different concentrations of THSWD serum and the inhibitor lonafarnib. The lgIC50=Xm-I(P-(3-Pm-Pn)/4). In MCF-7 cells, the IC50 dose of THSWD was determined to be 21.7% for 48 h, while for the inhibitor Lonafarnib the IC50 was 20.9 μM for 48 h. In MDA-MB-231 cells, the IC50 dose of THSWD was 19.5% for 48 h, while for the inhibitor lonafarnib the IC50 was 19.9 μM for 48 h (**Figure 9**).

#### Effects of THSWD Serum on the mRNA Expression of HRAS, MAPK1, AKT1, GRB2, and MAPK14 in Breast Cancer Cells

**Figure 10:**
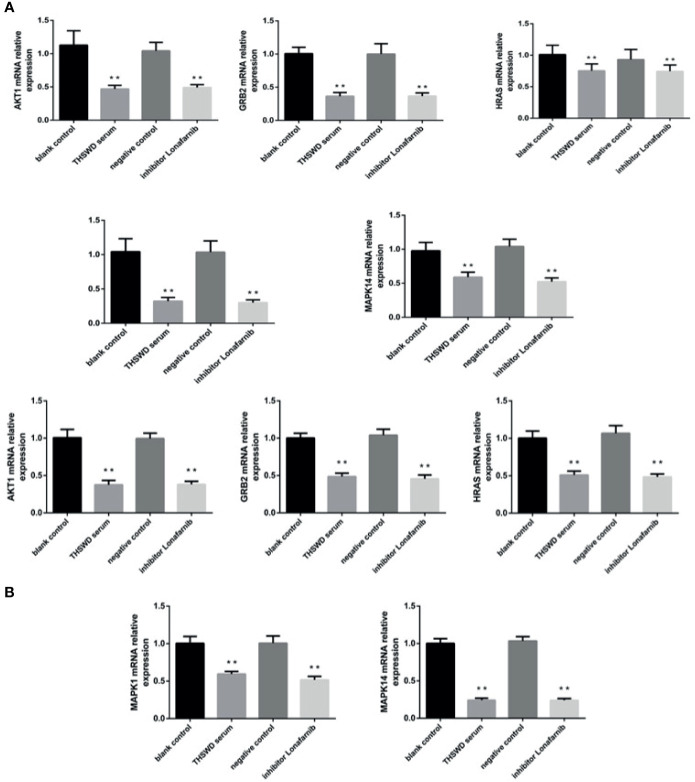
The expression of breast cancer-related genes in MCF-7 and MDA-MB-231 cells after treatment with THSWD **(A)** MDA-MB-231 **(B)** MCF-7. **P < 0.01 compared with blank control group.

The network pharmacology results identified potential key targets and pathways of THSWD active against breast cancer. To verify the reliability of the network pharmacology prediction results, mRNA levels of these key genes (HRAS, MAPK1, AKT1, GRB2, and MAPK14) were assessed by qRT-PCR in MCF-7 and MDA-MB-231. As shown in **Figure 10**, 48 h after the cell lines in each group were treated, the mRNA levels of HRAS, MAPK1, AKT1, GRB2, and MAPK14 were significantly down-regulated in THSWD serum and inhibitor-treated groups compared with controls and normal serum groups, respectively (P<0.01). The results suggested that THSWD serum and inhibitor could inhibit HRAS, MAPK1, AKT1, GRB2, and MAPK14 transcript levels.

#### Effects of THSWD Serum on Protein Expression of HRAS, MAPK1, AKT1, GRB2, and MAPK14 in Breast Cancer Cells

**Figure 11:**
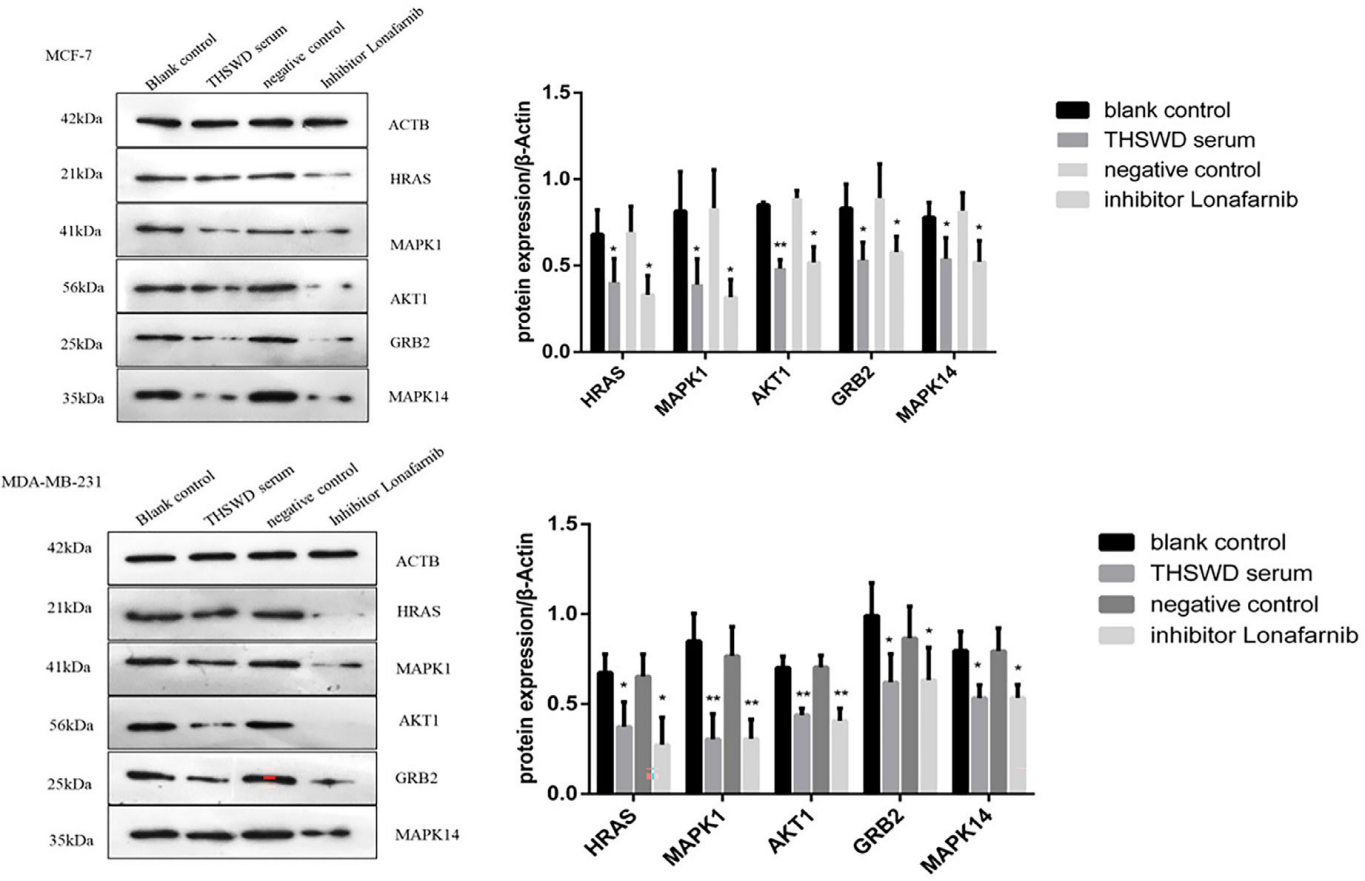
Effect of serum containing THSWD on the protein expression of HRAS, MAPK1, AKT1, GRB2 and MAPK14 in MCF-7 and MDA-MB-231 cells. *P < 0.05, **P < 0.01 compared with blank control group.

Western blotting analysis was used to examine the expression of HRAS, MAPK1, AKT1, GRB2, and MAPK14 proteins in MCF-7 and MDA-MB-231. As shown in **Figure 11**, based on triplicate experiments, the levels of HRAS, MAPK1, AKT1, GRB2, and MAPK14 were slightly decreased in THSWD serum compared to the controls and the normal serum group. The results showed that THSWD could down-regulate the protein levels of HRAS, MAPK1, AKT1, GRB2, and MAPK14 in MCF-7 and MDA-MB-231 cells.

## Discussion

Surgical resection, radiotherapy, and chemotherapy are the three most common therapies for clinical breast cancer treatment; however, surgical resection cannot improve prognosis, and long-term radiotherapy and chemoradiotherapy can cause severe toxic effects. THSWD is a combination of the classic prescription Siwu Decoction plus Prunus persica (L.) Batsch and Carthamus tinctorius L. THSWD can comprehensively exert it drug activity using multiple mechanisms. It is currently used clinically as an adjunctive treatment of diseases such as breast cancer and has good efficacy for improving the hypercoagulable state of blood and preventing thrombosis in cancer patients (15). In this study, in order to explore the therapeutic mechanism of THSWD in breast cancer, network pharmacology was used to explore the key pharmacodynamic agents and target pathways of THSWD activities for breast cancer treatment by constructing “ingredients-targets,” “ingredients-targets-pathway” and other networks; PPI, GO functional enrichment, KEGG pathway enrichment and molecular docking of key active ingredients and key targets were performed.

In total, 27 active ingredients and 218 common targets were used to construct the “ingredient-target” network. The results indicated that 11 compounds including luteolin, kaempferol, and senkyunolide E might be the key ingredients in THSWD able to exert therapeutic effects against breast cancer. Luteolin is a natural flavonoid, significantly inhibited proliferation and suppressed the expression of p-STAT3, p-EGFR, p-Akt, and p-Erk1/2 in EGF-treated MCF-7 breast cancer cells (16), it also inhibited proliferation and Notch signaling-associated protein expression and regulated miRNAs in MDA-MB-231 human breast cancer cells (17). Found that combination treatment with luteolin and celecoxib in MCF-7 and MCF7/HER18 cells disturbed cell progression through the G1 phase, enhanced the expression of death receptors (such as DR5), and activated the caspase cascade. Instead, luteolin could increase Bax expression by inhibiting Bcl-2 expression, enhancing mitochondrial membrane potential collapse, and cytochrome c release (18). In MCF-7 breast cancer cells treated with 12-O-tetradecanoylphorbol-13-acetate (TPA), luteolin suppressed the expression of interleukin 8 (IL-8) and the activation of matrix metalloproteinase 9 (MMP-9), which play important roles in breast cancer proliferation. Luteolin inhibits mRNA expression by inhibiting the mitogen activated protein kinase (MAPK) signaling pathway and down regulating the AP-1 and NF-κB. In addition, luteolin inhibited TPA-induced ERK1/2 phosphorylation, and inhibited the ERK1/2 pathway following IL-8 and MMP-9 expression (19). Kaempferol is a natural flavonoid widely distributed in nature. Modern pharmacological studies have revealed that kaempferol has anti-tumor, anti-oxidant, and anti-inflammatory effects (20), and exhibits suppressive effects on a variety of tumors, including gastric (21), esophageal (22), and breast cancers (23). Kaempferol treatment of MDA-MB-231 cells for 48 h resulted in a significant decrease in the number of cells in the G1 phase, from 85.48% to 51.35%, and a significant increase in the number of cells in the G2 phase, from 9.27% to 37.5%, indicating that kaempferol contributes to the induction of G2/M arrest, and can also induce apoptosis and DNA damage in MDA-MB-231 cells. Senkyunolide E belongs to the phthalides class of compounds with anti-inflammatory efficacy (24) and is speculated to be associated with modulating the tumor microenvironment.

KEGG pathway enrichment results indicated that THSWD might exert its therapeutic effects on breast cancer by regulating Ras, FoxO, and PI3K-Akt signaling pathways, which have been confirmed to be involved in breast carcinogenesis. Of these, the Ras pathway is the most significant pathway, Ras oncogenes are the most common oncogenes in human cancer, members of this superfamily of GTPases (KRAS, NRAS, and HRAS), which encode four highly conserved Ras proteins sharing 85% homology. Ras protein activity is regulated by binding to GTP or GDP and involves three main downstream signaling pathways: Ras/Raf/ERK, Ras/PI3K/AKT, and Ral-GEF (25). Based on the enrichment results, THSWD exerted its therapeutic effects mostly *via* Ras/Raf/ERK, Ras/PI3K/AKT, while both pathways were closely related to cell apoptosis and proliferation (26, 27).

The Ras-MAPK signaling pathway is involved in a variety of human tumors and development processes. MAPK signaling follows a tertiary enzymatic cascade, the Ras-Raf-MEK-MAPK pathway. Four subfamilies have been identified in the MAPK pathway, of which the extracellular signal regulated protein kinase (ERK) is the most studied. The main mechanism currently recognized for the Ras-MAPK/ERK signaling pathway is that this pathway, once aberrantly activated, auto mutation activation and sustained activation by upstream signaling, further activates downstream proteins, leading to cell proliferation, vascularization, apoptosis inhibition, tissue invasion, and ultimately promotes tumor development. The Ras-MAPK pathway can also act as a tumor suppressor by inducing cell senescence and apoptosis, and as a MAPK subfamily, active ERK is abnormally elevated in breast cancer cells during development and progression (28), aberrant ERK activation leads to proliferation and apoptosis inhibition in breast cancer (29).

Through *in vitro* experiments, using THSWD serum as an intervention, we selected two types of breast cancer cell lines, MCF-7 and MDA-MB-231, to conduct CCK-8 experiments, and found that THSWD exerted inhibitory effects on the proliferation of breast cancer cells, and the optimal concentration of THSWD serum was approximately 20%. Western blotting and PCR experiments proved THSWD induced down-regulation of HRAS, MAPK1, AKT1, GRB2, and MAPK14 protein and mRNA levels in two types of cell lines. These targets were the top five targets identified in the previous PPI network analysis, MAPK is mitogen activated protein kinases, a family of serine/threonine protein kinases, that are important transmitters of signals from the cell surface to the interior of the nucleus, and can transduce extracellular signals into the nucleus, through cascades that phosphorylate and activate downstream transcription factors, to regulate gene expression, and ultimately participate in various physiological processes, such as cell invasion, differentiation, proliferation, and apoptosis (30). MAPK1 is phosphorylated by upstream kinases and upon activation, it translocates to the nucleus of stimulated cells where it phosphorylates nuclear targets. MAPK1 has been shown to be involved in processes such as autophagy, lipid metabolism, proliferation, migration, and invasion (31, 32), HRAS belongs to the RAS family of small GTPases and is a frequently mutated oncogene in cancer, HRAS regulates a complex signal transduction network, including the RAF-MEK-ERK cascade, VEGF-PI3K-AKT pathway and Raf-1 signaling to promote cancer cell proliferation, migration, angiogenesis, and autophagy (33).

Thus, our findings show that THSWD regulates breast cancer in many ways through active ingredients and targets. THSWD can suppress breast cancer cell proliferation. Therefore, these results provide a valuable theoretical basis for THSWD as a potential drug for the treatment of breast cancer.

## Conclusions

In the present study, the network pharmacology approach was adopted for the first time to explore the underlying mechanism of THSWD on breast cancer. Studies on the MCF-7 and MDA-MB-231 cells showed that THSWD had significant anti-cancer activities. By network pharmacology analysis, the results demonstrated that the anti-cancer mechanism of THSWD might be through modulation of the Ras, FoxO, and PI3K-Akt signaling pathways. THSWD serum regulated the expression of cancer-related genes and proteins. It induced apoptosis, and inhibited cell proliferation of MCF-7 and MDA-MB-231 cells. The anti-cancer effect of THSWD might by achieved *via* the down-regulation of MAPK1, HRAS, GRB2, AKT1, and MAPK14. Our study demonstrated the reliability of the network pharmacology method, as well as revealed the anti-cancer effect and potential mechanisms of action of THSWD.

## Data Availability Statement

The datasets presented in this study can be found in online repositories. The names of the repository/repositories and accession number(s) can be found in the article/supplementary material.

## Ethics Statement

The animal study was reviewed and approved by The Committee on the Ethics of Animal Experiments of Anhui University of Chinese medicine (Permit Number: AHUCM-Rats-2021023).

## Author Contributions

SH and YC contributed equally to this work. SH and YC conceived and designed the study. SH, LP, CF, NW, and FC performed the *in vitro* experiments. SH and YC wrote the manuscript. YW and XC provided ideas for the experimental design and modified the manuscripts to ensure the integrity of the entire experimental design. All authors contributed to the article and approved the submitted version.

## Funding

This research was supported by the National Natural Science Fund Regional Innovation and Development Joint Fund Project (No.U19A2009), National Natural Science Foundation of China (Grant No. 82074059), Anhui University Collaborative Innovation Project (GXXT-2019-043), the Anhui Provincial College Natural Science Research Key Project (No. KJ2019A0466), Excellent and Top Talents Program in Colleges and Universities (No. gxyq2019034), Anhui Provincial Key Laboratory of Traditional Chinese Medicine Compounds (2019AKLCMF03), and the Natural Science Research Project of Colleges and Universities in Anhui Province (2019fyyb038).

## Conflict of Interest

The authors declare that the research was conducted in the absence of any commercial or financial relationships that could be construed as a potential conflict of interest.

## Publisher’s Note

All claims expressed in this article are solely those of the authors and do not necessarily represent those of their affiliated organizations, or those of the publisher, the editors and the reviewers. Any product that may be evaluated in this article, or claim that may be made by its manufacturer, is not guaranteed or endorsed by the publisher.
